# Felt Hats

**Published:** 1856-04

**Authors:** 


### FELT HATS.

The tyranny of fashion presses heavily on the heads of men and women now-a-days. There is the bonnet placed so far back on the head that it occasions an abiding feeling as if it were going to fall off, and ladies are seen every few minutes throwing their chins up and their heads back as if a bug were crawling along the back and they wished to scratch it. No doubt the fashion was devised by some hump-shouldered beauty or heiress to hide her deformity, and anon, beauty and gold made it "*the rage.*" But we are free to say that the fashion never troubles us, for it's the beauty, not the bonnet which entrances our eyes.

As to the head gear of the gentlemen, the persistence they exhibit in wearing the cumbersome, hard, paste-board hats, covered with silk, instead of the soft, light, pliable, cool "*Felt Hat*" which a few only, as yet, have been bold enough to wear, we say, the persistence in submitting to the silk hat tyranny would be incredible, were it not a matter of daily observation.

The silk hat when first worn, not only feels much as if an iron hoop were circling the head, but it leaves an ugly, red streak around the forehead, reminding us, by its color, of a boiled lobster, to say nothing of the headache which it originates, almost as often as it is long worn, for the several weeks or months which are required to adapt itself to the soft head which wears it.

We venture to say that no man would from simple choice ever wear a silk hat after having worn the pliable *Felt* for a day or two. The broadness of their brims affords a necessary and most complete protection to the eyes and face against the glare and tanning influence of the sun light. Will the Messrs. Leary & Co., Astor House, Broadway, who make so superior an article send one up this way for so favorable a notice of their manufacture?

To clergymen, the circulation about whose heads should be always free and full, as to blood and air, the Felt hat is a desideratum, while it imparts a patriarchal look which much becomes their calling.

